# Patterns and predictive factors of loss of the independence trajectory among community-dwelling older adults

**DOI:** 10.1186/s12877-021-02063-7

**Published:** 2021-02-26

**Authors:** Charlotte Bimou, Michel Harel, Cécile Laubarie-Mouret, Noëlle Cardinaud, Marion Charenton-Blavignac, Nassima Toumi, Justine Trimouillas, Caroline Gayot, Sophie Boyer, Réjean Hebert, Thierry Dantoine, Achille Tchalla

**Affiliations:** 1 CHU de Limoges, Pôle HU Gérontologie Clinique, Service de Médecine Gériatrique, Unité de Prévention de Suivi et d’Analyse du Vieillissement (UPSAV), CHU Limoges, 2 Avenue Martin-Luther King, F-87042 Limoges, France; 2 Université de Limoges; EA 6310 HAVAE Handicap Activité Vieillissement Autonomie Environnement, F-8705 Limoges, France; 3 Unité de Recherche Clinique et de l’Innovation en Gérontologie (URCI), Hôpital Jean Rebeyrol, CHU de Limoges, 87042 Limoges, France; 4 HAVAE Laboratory, University of Limoges, 123 avenue Albert Thomas, F-87060 Limoges, France; 5 Institut de Mathématiques de Toulouse et École Supérieure du Professorat et de l’Éducation à l’Université de Limoges, 87000 Limoges, France; 6 Université de Montreal, Québec, Canada

**Keywords:** Independence, Functional decline, Prevention, Trajectory, Older adults, Semi-parametric model, Optimal number of groups

## Abstract

**Background:**

Independence is related to the aging process. Loss of independence is defined as the inability to make decisions and participate in activities of daily living (ADLs). Independence is related to physical, psychological, biological, and socioeconomic factors. An enhanced understanding of older people’s independence trajectories and associated risk factors would enable the develop early intervention strategies.

**Methods:**

Independence trajectory analysis was performed on patients identified in the Unité de Prévention de Suivi et d’Analyse du Vieillissement (UPSAV) database. UPSAV cohort is a prospective observational study. Participants were 221 community-dwelling persons aged ≥75 years followed for 24 months between July 2011–November 2013 and benefits from a prevention strategy. Data were collected prospectively using a questionnaire. Independence was assessed using the “Functional Autonomy Measurement System (Système de Mesure de l’Autonomie Fonctionnelle (SMAF))”. Group-based trajectory modeling (GBTM) was performed to identify independence trajectories, and the results were compared with those of k-means and hierarchical ascending classifications. A multinomial logistic regression was performed to identify predictive factors of the independence trajectory.

**Results:**

Three distinct trajectories of independence were identified including a “Stable functional autonomy (SFA) trajectory” (53% of patients), a “Stable then decline functional autonomy decline (SDFA) trajectory” (33% of patients) and a “Constantly functional autonomy decline (CFAD) trajectory” (14% of patients). Not being a member of an association, and previous fall were significantly associated of a SDFA trajectory (*P* < 0.01). Absence of financial and human assistance, no hobbies, and cognitive disorder were significantly associated with a CFAD trajectory (*P* < 0.01). Previous occupation and multiple pathologies were predictive factors of both declining trajectories SDFA and CFAD.

**Conclusions:**

Community-living older persons exhibit distinct independence trajectories and the predictive factors. The evidence from this study suggests that the prevention and screening for the loss of independence of the older adults should be anticipated to maintaining autonomy.

## Background

According to National Institute for Statistics and Economic Studies (Institut national de la statistique et des études économiques (INSEE)), French older adults population aged ≥75 years is expected to reach more than 11.9 million and those aged ≥85 years to reach more than 5.4 million in 2060 [3]. This aging would be accompanied by chronic diseases, physical, psychological, biological, and socioeconomic difficulties, dementia that can lead to a loss of independence and institutionalization. Loss of independence is associated with aging, as is disability [45], and can be defined as the inability make decisions and/or perform activities of daily living. With the aging population growing rapidly, the number of dependent people is increasing [11, 25]. Several tools developed to assess older person independence degree including: activities of daily living (ADL) [28], instrumental activities of daily living (IADL) [29], the Independence Gerontology Iso-Resource Groups (AGGIR) grid [50], a system for measuring functional independence (SMAF) [21, 22], and the multidimensional evaluation guide Resident Assessment Instrument (RAI) [20]. These tools are available or under evaluation in France and in other nations for assessing the needs of older people who have lost their independence.

In this study, we investigated the patterns of independence loss in a representative sample of French community-dwelling adults aged ≥75 years using the SMAF tools. Most prior studies of the independence trajectories of older adults used the ADL [26] or GIR [8] score, and those that did use SMAF were descriptive analysis [10, 21–23]. The SMAF tool was developed in Canada in 1984 ([16, 21, 22], and comprises 29 functions in five categories: ADLs, mobility, communication, mental functions, and IADLs. Each function is evaluated on a fifth-point scale, as follows: 0 (independence), 0.5 (difficulty), 1 (need for stimulation or supervision), 2 (assistance), 3 (complete help or dependence) [21, 22]. The SMAF is available in multiple languages and is used in the clinical setting in, for example, Canada (Quebec) and France. Its validity and reliability have been verified.

As part of the longitudinal follow-up of the UPSAV cohort, we investigated the independence trajectories of older adults residing in their own homes. The UPSAV is an innovative system initiated in France and aimed at preventing the global disruption of the older adults’ autonomy and assessing the health, social and economic impact of preventive measures. Early identification of older person at risk of decline functional autonomy is important for delivering preventive interventions. The aim of this study was to identify older adults who would benefit from the UPSAV intervention.

## Methods

The method described in this paper refers to Bimou’s thesis [1].

### Study design and population

Participants were members of the UPSAV prospective and longitudinal study of 221 conducted from July 2011 to November 2013 among community- living persons, aged over 75 years in Limousin, France. Each participant was followed for 2 years and was assessed by a geriatrician at 0, 6, 12, and 24 months. Our time variable (T0, T1, T2, T2, T3) corresponds to the four visits. The inclusion criteria were: age ≥ 75 years, registration with the social security system, complementary health or 100% coverage by social security, and the intellectual capacity to understand the protocol and submit to the interventions or mild to moderate dementia (Mini Mental Test Statement [MMSE] score ≥ 10). Also, the patient or their legal representative must have provided written informed consent. After inclusion, the study patients have benefited a comprehensive geriatric assessment. An intervention plan was established and coordinated by the UPSAV. Thus, the study participants benefited from a prevention strategy during follow-up time. The data were collected through questionnaires.

### Assessment of functional autonomy

The SMAF is an indicator used to predict the transitions of Iso-SMAF profiles over 4 years with 1500 people over the age of 75 followed annually cohort PRISMA [39]) and examined covariates related to transitions of autonomy [40]. The PRISMA is the research group established to address the problem of lack of continuity to care experienced by older adults with chronic conditions in Quebec. Its objective was to evaluate the implementation of an Integrated Service Delivery Network (ISD French acronym) to improve the health, empowerment and satisfaction of frail older people and to change health and social service utilization without increasing caregiver burden [24]. SMAF is a quantitative variable ranging from 0 to 87 points [21–23]. We chose this variable because it has never been the participant of a study of older adults autonomy trajectories and it is quite complete compared to other tools [10]. Based on epidemiological data and on the observation of the distribution of ISO-SMAF Profiles [10], a SMAF score between 0 and 7 indicates complete autonomy, between 8 and 14 we speak of average autonomy. A SMAF score ≥ 15 was determined to be the best descriptor of moderate to severe loss of autonomy.

### Potential predictive factors

The explanatory variables considered as potential predictive factors of independence trajectory included sociodemographic: age, sex, occupation, educational level, place of housing, type of housing, place of residence, marital status, lifestyle, monthly income, financial, human and technical assistance, hobbies, association membership. The health-related variables were: comorbidities [9], daily medications [37], urinary incontinence, anal incontinence, visual disorder, auditory disorder, and communication disorder. Cognitive ability measures consisted the Mini-Mental State Examination (MMSE) [6, 27, 37]. The total MMSE score is 30 points; a score of < 18 is defined as moderate or severe cognitive impairment [6]. We used the Cognitive Evaluation Reflection Group (GRECO) standards to dichotomize the MMSE scores; suspected dementia was defined as an MMSE score of < 24 [27]. Depressive state was evaluated using the 30-points Geriatric Depression Scale (GDS). The GDS scores were classified as: 0–9 no depression, 10–19 mild depression, 20–30 severe depression [5]. Nutritional status included the Mini Nutritional Assessment (MNA) [49], serum albumin level (Guigoz, 1997), body mass index (BMI). An MNA score of ≥24 is defined as an adequate nutritional status; an MNA score of 17–23.5 as risk of malnutrition, and an MNA score of < 17 is regarded as indicative of protein malnutrition [49]. In this study we defined a good and poor nutritional status as an MNA score of ≥24 and < 24, respectively. A serum albumin level of < 30 g /L was defined as a poor nutritional status. The body mass index (BMI) (kg/m^2^) was calculated by dividing the weight by the square of the height in meters. There are no standards for the interpretation of the BMI of older persons [31]. Nevertheless, obesity is generally defined as a body mass index (BMI) of 30 kg/m2 and higher. Overweight is defined as a BMI between 25 and 30 kg/m2 [42]. We categorized the subjects’ BMI as < 20 (abnormal weight), 21–24 (normal weight), or > 25 (excess weight). Fragility variables included Fried test [12, 13], 12-point Physical Performance Battery (SPPB) scale [18], fall during the previous year and unipodal support test [49]. A score of 0–6 indicates low physical performance, 7–9 average performance, and a score of 10–12 indicates good physical performance [18]. A unipodal support test result of < 5 s was regarded as indicative of an equilibrium disorder. Table 1 provides detailed overview of those variables.

**Table 1:** Characteristics of the Study Population

Sociodemographic Characteristics and Risk Factors	Total sample *n* = 221^a^	
Age (mean, SD^b^) 86.1, ±5	No.	%
Age		
≥ 80 years	142	64.25
< 80 years	79	35.75
Sex		
Woman	149	67.42
Man	72	32.58
Profession		
Trader/Liberal professional	47	21.27
Public Service/Executive/Intermediate Occupation	45	20.36
Employee/Intermediate profession in company	41	18.55
Housewife, Other occupation	32	14.48
Worker	21	9.50
Executive manager/entrepreneur	19	8.6
Farmer	16	7.24
School level		
Certificate of Primary Education	84	38.01
Secondary/higher education	78	35.29
College certificate	35	15.84
Can read, write, count	24	10.86
Type of dwelling		
House	160	72.40
Apartment/Household	61	27.60
Geographical situation		
Urban	121	54.75
Rural	100	45.25
Family situation		
Widower	118	53.39
Married	82	37.10
Single/Divorced/Free Union	21	9.50
Lifestyle		
Single	130	58.82
In a couple / With a family member	91	41.18
Family Support		
Assistance	98	47.12
No assistance	110	52.88
Neighbor support		
Assistance	122	55.20
No assistance	99	44.80
Place of residence		
Owner	130	58.82
Beneficial owner	53	23.98
Tenant	38	17.19
Revenues		
≥ 2000 €	77	34.84
Between1500 and 2000 €	64	28.96
Between1000 and 1500 €	53	23.98
< 1000 €	27	12.22
Financial assistance		
Not existing	162	76.42
Existing	50	23.58
Human assistance		
No	128	57.92
Yes	93	42.08
Technical assistance		
No	158	94.05
Yes	10	5.95
Hobbies		
Yes	207	93.64
No	14	6.36
Member of an association		
No	119	54.59
Yes	99	45.41
Comorbidity≥2		
Yes	194	87.78
No	27	12.22
Number of drugs per day> 4		
Yes	169	76.47
No	52	23.53
Urinary incontinence		
No	131	59.28
Yes	90	40.72
Anal incontinence		
No	211	95.48
Yes	10	4.52
Visual disorder		
Yes	209	94.57
No	12	5.43
Hearing disorder		
No	112	50.68
Yes	109	49.32
Communication disorder		
No	221	100
Yes	0	0
MMS		
≥ 24	182	82.73
< 24	38	17.27
GDS		
< 9	133	60.45
≥ 9	87	39.55
MNA		
≥ 24	177	80.09
< 24	44	19.91
BMI, kg/m^2^, ^c^		
≥ 21	202	92.66
< 21	16	7.34
Albuminemia, g/L, ^d^		
≥ 35	199	91.71
< 35	18	8.29
Exhaustion		
< 20%	157	71.36
> 20%	63	28.64
Walking speed on 4.5 m,		
> 20%	176	79.64
< 20%	45	20.36
Endurance		
Good	158	71.49
Poor	63	28.51
Sedentary life		
No	143	64.71
Yes	78	35.29
Involuntary weight loss > 4.5 kg in the past year,		
No	204	92.31
Yes	17	7.69
SPPB		
Reduced performance	124	56.36
Good physical performance	52	23.64
Intermediate performance	44	20
Frailty index?		
Pre-frailty	135	61.09
Fragile	49	22.17
Robust	37	16.74
Antecedent of Fall		
Yes	136	61.54
No	85	38.46
Unipodal support < 5 s, ^e^		
No	122	55.20
Yes	99	44.80

^a^One of the patients was missing data and so was excluded from the analysis

^b^Standard deviation

^c^*BMI* weight in kilograms divided by height in meters squared

^d^Albuminemia was calculated as described previously [18]

^e^One-leg balance (ability to stand on one leg unassisted for 5 s) [17]

### Statistical analyses

Group-Based Trajectory Model (GBTM) [36] was used to identify latent trajectory groups for SMAF from scores between 0 to 87. GBTM is a particularity of finite mixture modeling. The method consists to cluster individuals into meaningful subgroups that show statistically similar trajectories [34, 35]. A statistical method is used to identify groups of distinctive trajectories which are summarized by a finite set of different polynomial functions of time. In our case, time is equal to visits. The complexity of estimating the parameters of the GBTM model requires maximization by the quasi-Newton procedure. The nature of the dependent variable SMAF (normal distribution) brought us to use the censored normal model [36]. Group’s trajectory, the form of each trajectory, are predicted. The probability for each individual of group membership is estimates. Which allow to assign them to the group for which they have the highest probability. Bayesian information criterion (BIC) criterion was used to select model [36]. We estimated seven models and selected the best model using the BIC. Missing data is a common drawback that appears in many real-world situations as in surveys. In our study, the lack of data was completely random and independent of the variable itself and any other external influences. For example, for the main variable SMAF, it was approximately 24% missing data in T1, 28% in T2 and 22% in T3. We used the multiple imputation method to manage missing data. Missing data were managed utilizing multiple imputation, which identifies missing values by performing repeated simulations [30]. We used PROC MI “multiple imputation procedure” in SAS to manage them. A multinomial logistic regression analysis was performed to analyze the dependence of the explanatory variable and to identify predictive factors. The final model was selected bases on the Bayesian information criterion [36]. The alpha level was set at 0.05.

Two other classification methods were used to identify trajectories. It is about k-means for longitudinal datasets (Kml) [14, 15] and hierarchical ascending classification (HAC). For k-means method, we used the Calinski-Harabasz criterion [7] to identify the optimal number of trajectory groups. Calinski-Harabasz criterion combines the within and between matrices to evaluate clustering quality. We used the “Kml” package in R software (v. 3.4.1; Core Team (2014) R Foundation for Statistical Computing, Vienna, Austria; http://www.R-project.org/) [15]. We used Ward’s aggregation criteria [51] to identify the optimal number of groups for the hierarchical ascending classification. Ward’s criteria consist to minimize intragroup inertia and maximize intergroup inertia. The method was implemented in R software.

Despite the application of those three methods, in this proposal, GBTM is the principal method because it is simple to implement, useful for describing the heterogeneity of SMAF scores evolution, identifying the risk factors, and potentially for informing clinicians about patients’ subgroups who would need more attention to maintain their functional autonomy. According to Twisk [48], GBTM was shown to be superior for identifying underlying longitudinal trajectories. The k-means and hierarchical ascending classification were performed to compare the optimal number of trajectory groups with the GBTM. Thus, for k-means and hierarchical ascending classification, we presented only the results of the trajectory groups. The results of Baseline characteristics and the logistic regression are based on the GBTM method. These methods are more detailed Bimou and colleagues’ study [2].

## Results

### Overall description of the study sample

Table 1 summarizes the description of the study sample at baseline. Variables including occupation, educational level and monthly income had rare modalities that were grouped together. The participants mean age were 86.1 ± 5.0 years old; About 64% of the participants were > 80 years old. Most study participants were female, resided in an urban area, had hobbies, no cognitive disorders and not depressive symptoms, whereas a relatively small minority had significant loss of weight and low monthly income.

### Application of BIC, Calinski-Harabasz, and Ward criterion

The BIC’s values, Calinski-Harabasz’s and Ward’s criteria are listed in Table 2. GBTM results showed a fairly significant decrease between the first model (k = 2, BIC = − 3229) and the second model (k = 3, BIC = − 2424); 14.5% of participants were classified into the smallest subgroup in first model, compared to 8.9% in second model. Calinski-Harabasz’s criterion decreased from 378 (k = 3) to 317 (k = 4), subsequently increased rapidly from k = 4, and thereafter decreased. Ward’s criterion provided a large jump of inertia between k = 2 and k = 3. Inertia value begins to stabilize when the group number exceeds three. Thus, the best-adapted models included three groups of independence trajectories.

**Table 2:** BIC, Calinski-Harabasz Criterion, and Hierarchical Ascending Classification Criterion Values and Predicted Proportions of the Group-Based Trajectory Models

					Number of patients by group (%)					
**GBTM**	**Models**	**Groups, k**^**b**^	**BIC**	**1**	**2**	**3**	**4**	**5**	**6**	**7**
	1	2	-3229	66.1	33.9	–	–	–	–	–
	2	3	-2424	53	32.5	14.5	–	–	–	–
	3	4	− 2669	39.9	28.8	22.2	8.9	–	–	–
	4	5	− 2615	18.3	31.9	20.3	20.7	8.7	–	–
	5	6	− 2579	17.1	30	21	17.6	10.5	3.6	–
	6	7	− 2595	15	25.7	16.4	12.9	16.1	10.2	3.4
**K-means**	**Models**	**Groups, k**^**b**^	**Calinski-Harabasz criterion**							
	1	2	402	58.4	41.6	**–**	**–**	**–**	**–**	**–**
	2	3	378	35.7	38	26.2	–	-	–	–
	3	4	317	33.5	28.9	13.6	24	–	–	–
	4	5	351	33	28.9	13.1	1.4	23.5	–	–
	5	6	313	34.8	27.1	6.3	6.8	16.7	8.1	–
	6	7	296	11.3	23.1	2.3	7.7	25.8	9.5	20.4
**HAC**^**a**^	**Models**	**Groups, k**^**b**^	**Ward criterion**							
	1	2	16	70.6	29.4	**–**	**–**	**–**	**–**	**–**
	2	3	10	59.7	27.6	12.7	–	–	–	–
	3	4	6	59.3	25.4	12.7	2.7	–	–	–
	4	5	5	33	26.7	17.6	19.5	3.2	–	–
	5	6	5	32.1	28.5	16.7	7.7	12.2	2.7	–
	6	7	4	32.1	28.5	16.7	6.8	8.1	5.4	2.3

^a^Hierarchical ascending classification

^b^k, number of groups

### Patterns of Independence trajectories

**Fig. 1:**
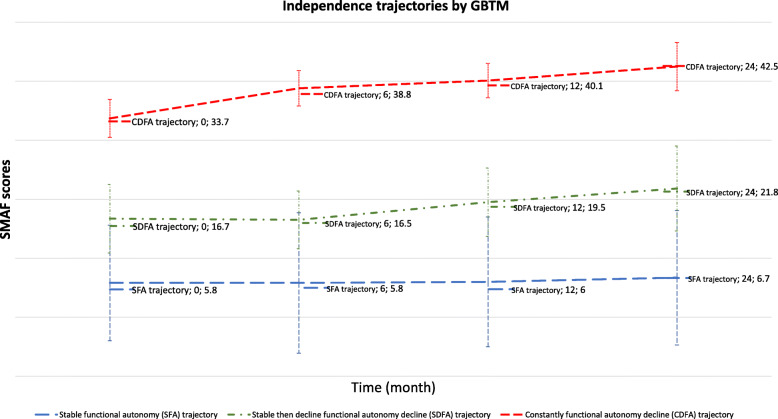
Trajectories of independence determined using the GBTM method among 221 subjects aged > 75 years in France from 2011 to 2013, benefiting a prevention strategy during the follow-up time. The best model based on the BIC value (−2424, *n* = 221) was the three groups model

**Fig. 2:**
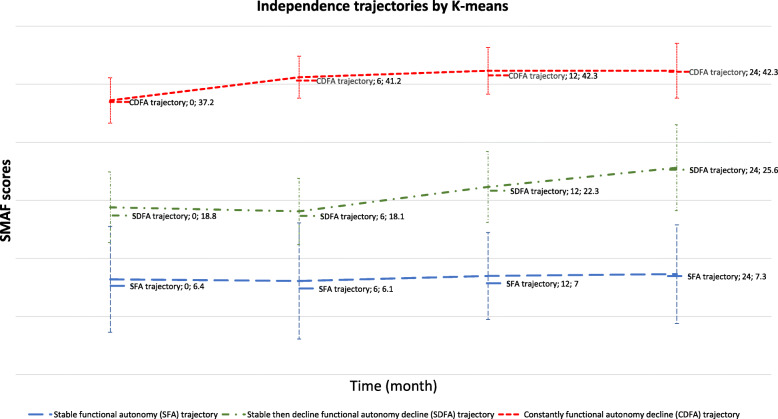
Trajectories of independence determined using the k-means method among 221 subjects aged > 75 years in France from 2011 to 2013, benefiting a prevention strategy during the follow-up time. The best model based on the Calinski-Harabasz value (378, *n* = 221) was the three groups model was the three groups model

**Fig. 3:**
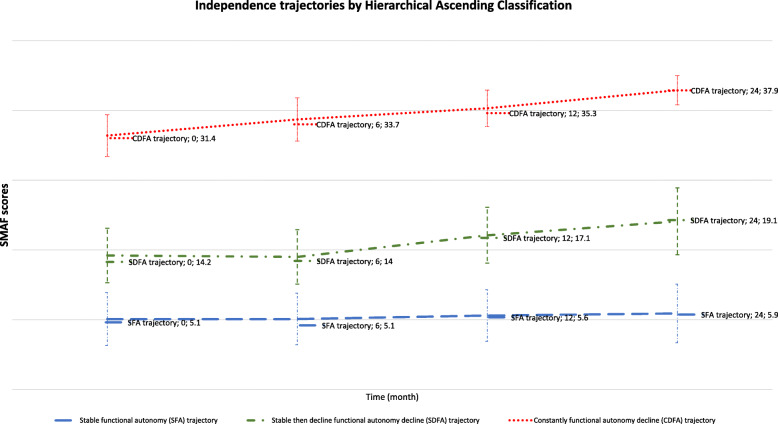
Trajectories of independence determined using the HAC method among 221 subjects aged > 75 years in France from 2011 to 2013, benefiting a prevention strategy during the follow-up time. The best model based on the Ward’s jump value (10, *n* = 221) was the three groups model

Figures 1, 2 and 3 show the three trajectory groups formed by the three methods. Among the seven models performed, only the two- and three-group models converged for GBTM method (Fig. 1). Therefore, we selected the three-group model for further analysis. Similarly, in the k-means and hierarchical ascending classifications, the model comprising three independence trajectory groups best fit the data.

The GBTM model comprising three groups showed a posterior probability of 0.73 ± 0.14 to 0.98 ± 0.17. The three groups were: Stable functional autonomy trajectory (SFA) (*n* = 117, average SMAF score between 5.8 and 6.7, 53%, highly independent older adults), Stable then decline functional autonomy decline trajectory (SDFA) (*n* = 72, 33%, average SMAF score 16.7–21.8, older adults with moderate-to-severe dependence), and Constantly functional autonomy decline trajectory (CFAD) (*n* = 32, 14%, average SMAF score 33.7–42.5, dependent older adults). The three trajectory groups obtained by k-means and hierarchical ascending classification showed the similar groups those found by the GBTM and described in the same way. Thus, we obtained for k-means: SFA (*n* = 150, SMAF average 6.4–7.3, 67.9%), SDFA (*n* = 53, 24%, SMAF average 18.8–25.6), and CFAD (*n* = 18, 8%, SMAF average 37.2–42.4). Hierarchical ascending classification showed following groups: SFA (*n* = 136, 62%, average SMAF average 5.1–5.9), SDFA (*n* = 60, 27%, SMAF average 14.2–19.1), and CFAD (*n* = 25, 11%, SMAF average 31.4–37.9).

### Baseline variables related to the Independence trajectories

Table 3 provides the adjusted values of OR from multinomial logistic regression. Multinomial logistic regression revealed that specific baseline characteristics predicted membership within each of the three functional autonomy trajectory groups as compared to the Stable functional autonomy trajectory group. The predictive factors of Stable then decline functional autonomy decline trajectory were as follows: farmer (OR = 10.7, 95% CI = 1.09–14.44, *p* = 0.041), non-membership of an association (OR = 2.67, 95% CI = 1.02–7.00, *p* = 0.005), and a fall in the previous year (OR = 2.72, 95% CI = 1.28–5.77, *p* = 0.009). The predictive factors of a Constantly functional autonomy decline trajectory were: worker (OR = 10.33%, CI = 0.74–15.60, *p* = 0.081), lack of financial assistance (OR = 2.35, 95% CI = 0.09–7.56, p = 0.009), lack of human assistance (OR = 3.30, 95% CI = 0.03–8.26, *p* = 0.001), lack of hobbies (OR = 22.21, 95% CI = 1.44–34.25, *p* = 0.026), and cognitive disorder (OR = 2.12, 95% CI = 0.95–10.05, *p* < 0.0001). The previous occupation and multiple pathologies were predictive factors for both above trajectories.

**Table 3:** Baseline Factors Associated with Trajectory by Multinomial Logistic Regression Analysis Using the Stable-Low Trajectory Group as the Reference

Predictive factors	Stable-low then higher trajectory (*N* = 72, 33%)			Constantly higher trajectory (*N* = 32, 14%)		
	OR^a^	95%CI^b^	*P*-value^c^	OR^a^	95%CI^b^	*P*-value^c^
Age						
≥ 80 years	1.00	Referent		1.00	Referent	
< 80 years	0.25	0.10–0.66	0.005	0.19	0.02–1.37	0.10
Profession						
Housewife, Other profession	1.00	Referent		1.00	Referent	
Farmer	10.70	1.09–14.44	**0.041**	0.43	0.09–20.09	0.666
Worker	1.38	0.19–9.97	0.747	10.33	0.74–15.60	0.081
Employee/ Intermediate profession in company	1.47	0.33–6.36	0.606	0.10	0.05–2.25	0.150
Executive manager, entrepreneur	0.83	0.12–5.37	0.847	0.38	0.17–8.48	0.545
Trader/ Liberal profession	1.06	0.65–12.72	0.159	1.21	0.15–9.47	0.856
Employee/Senior/ Intermediate Public Service Occupation	0.60	0.40–8.24	0.435	2.26	0.21–24.42	0.499
School level						
Secondary/higher education	1.00	Referent		1.00	Referent	
Can read, write, count	5.30	0.96–9.16	0.055	3.01	0.25–36.42	0.384
Certificate of Primary Education	0.54	0.17–1.70	0.293	0.25	0.04–1.56	0.139
College certificate	0.40	0.11–1.46	0.166	0.19	0.02–1.73	0.142
Financial assistance						
No assistance	1.00	Referent		1.00	Referent	
Assistance	0.36	0.11–1.12	0.071	2.35	0.09–7.56	**0.009**
Human assistance						
No assistance	1.00	Referent		1.00	Referent	
Assistance	0.24	0.09–0.61	0.003	3.30	1.13–8.26	**0.002**
Hobbies						
No	1.00	Referent		1.00	Referent	
Yes	1.38	0.21–9.08	0.732	22.21	1.44–34.25	**0.001**
Membership of an association						
No	1.00	Referent		1.00	Referent	
Yes	2.67	1.02–7.00	**0.005**	1.05	0.25–4.37	0.056
Comorbidity> 2						
No	1.00	Referent		1.00	Referent	
Yes	3.79	1.48–9.68	**0.005**	4.89	0.95–25.05	0.0565
MMS						
≥ 24	1.00	Referent			Referent	
< 24	0.31	0.27–1.98	0.540	2.12	1.95–10.05	**<.0001**
MNA						
≥ 24	1.00	Referent		1.00	Referent	
< 24	0.35	0.12–1.04	0.056	1.31	0.51–3.28	0.5632
Antecedent fall						
No	1.00	Referent		1.00	Referent	
Yes	2.72	1.28–5.77	**0.009**	1.31	0.52–3.31	0.418

^a^*OR* odds ratio

^b^95% CI, 95% confidence interval. The probability that the estimates contain the parameter estimated with a margin of error of 5%

^c^Two-sided p-value

## Discussion

The main objective of this study was to identify trajectories of autonomy. The findings presented in this study show that GBTM, k-means, and HAC can be applied successfully to autonomy trajectories. The analysis advanced our knowledge of individuals analyzed behavior. It allows us to describe different subgroups of autonomy that follow specific trajectory over time. The results of the three models suggested that the optimal number of homogeneous groups of independence was three and analysis reveal three trajectories over 24 months following. Thus, the independence development studied using SMAF scores in older adults aged over 75 years old helped to identify three groups of older adults, following three trajectories of possible independence over four observation periods: a first group following a Stable functional autonomy trajectory, a second group following Stable then decline functional autonomy decline trajectory, and a third group following a Constantly functional autonomy decline trajectory.

The analysis shows that approximately a little more than a half of participants (117 participants, 53%) had high levels of functional independence upon inclusion that remained high across the independence trajectory as shown by the average SMAF values in Figs. 1, 2 and 3. The trajectory of the other half was consistently above the PRISMA threshold [10, 38]. The Figs. 1, 2 and 3 show more details of the values. Participants presenting high levels of independence represented autonomous participants. However, participants reporting low levels of independence represented dependent participants.

To our knowledge, no other study has used SMAF scores to estimate distinct trajectories of functional autonomy for longitudinal older adults’ data. Other studies, for example, that of [26] is in line with our results but based on ADL scores whose results suggest, or the Carrière’s study [8] based on AGGIR grid. Jonkman and al [26] works identified 3 distinct trajectories of functional decline over a 9-year follow-up using ADL scores. In the study [8], the author used the AGGIR grid to assess older adults’ independence and disability; some longitudinal studies used the 14 iso-SMAF profiles [4]. Our longitudinal study produced the first results using SMAF concerning independence trajectory analysis of older adults living in a community. This study has highlighted differences among older adults in Limousin regarding loss of independence identifying three distinct groups with different trajectories of independence.

Depending to the results of multinomial logistic regression, our study has highlighted differences in older adults’ trajectories of independence in terms of occupation and educational level. Belonging either to a moderately dependent older adults’ trajectory or to highly dependent older adults’ trajectory was influenced by sociodemographic and clinical variables. Some results suggest that the risk of becoming dependent depends not only on the state of health but also on factors related to sociodemographic characteristics such as age and educational level [4]. For example, the Sánchez-García study shows that schooling < 6 years is statistically associated with the presence of low autonomy in the older adults [41]. and his colleagues confirmed that the level of education would be associated with loss of independence in the older adults [32].

The ‘farmer’ category was associated with the stable then decline functional autonomy trajectory, and ‘worker’ with a constantly functional autonomy decline trajectory. This could reflect a differential presentation between this both people. A lack of hobbies would have an important impact on the stable then decline functional autonomy trajectory. According to Tomioka study, having neither hobbies was significantly associated with a decline ADL [47]. Membership of the constantly functional autonomy decline trajectory was associated with a lack of financial and human assistance, as well as non-membership in an association, as predictive factors of loss of independence. These findings are consistent with those of Xie, which suggest financial support for seniors [52]. Thus, some older people require professional assistance to participate in ADL. Our results indicate that older adults’ loss of independence would be linked to various sociodemographic factors.

Medical comorbidities were associated with higher dependence trajectories and contributed to the risk of loss of independence. According to Bressé [4], serious illnesses were found to be risk factors for the loss of independence. This shows a possible reinforce between the loss of functional autonomy and the health disorders. Cognitive impairment, and previous falls are predictive factors of loss of independence. Maria [44] reported that loss of independence as assessed using the ADL and IADL scores was a significant risk factor for cognitive deficit (MMSE < 16). Falls, which are frequently experienced by older people, are a major risk factor for loss of independence [43, 46].

## Limitations

This study has several limitations. First, the population small size (221 participants) and the short follow-up duration limit the generalizability of our results. But despite that, the multinominal logistic regression model predicts data with 85% accuracy. The results that we present here give a first idea of the trajectories. As a result, at this stage with the small sample size, our work is a first step but still with an explorative character. To generalize our results, in future research, we plan to assess the patterns observed in other older adult’s population monitored within the same as our population or the longer periods, a larger population.

Secondly, the functional autonomy of the participants was evaluated using the SMAF. Our results were interpreted using the SMAF independence threshold set by the PRISMA [38] and Dubuc [10] studies; i.e., a SMAF score of ≥16 indicated moderate-to-severe loss of independence. However, those study do not draw a distinction between those patients referred to as SMAF = 19 and those scored at 80. This inaccuracy could be a limitation in the interpretation of our study.

Thirdly, missing follow-up data are inevitable in geriatric studies, and may bias this analysis results. When the GBTM is used for trajectory analysis, non-random attrition of participants may affect the trajectory groups size [19], especially when groups are initially not well separated [33]. In our study, data attrition was mainly due to death and institutionalization. This concerned a minority of the participants. Nevertheless, data attrition may have led to biased estimates.

## Conclusion

In older people aged ≥75 years, 3 distinct trajectories of independence across 2 years of follow-up can be identified. The three trajectories did not evolve in the same way despite the UPSAV intervention. In geriatric practice, assessment of loss of autonomy is a crucial and unavoidable step because the purpose of geriatric intervention is to delay the onset of AHR dependence by preserving all or part of the autonomy, or even limiting its loss. Thus, UPSAV’s intervention consists of carrying out regular follow-up check-ups in the participant’s home. Our current data highlight that many older people in Limousin are stably independent, but the independence of a significant minority decreases over time. We identified various risk factors for the three independence trajectories; these can be used to formulate novel prevention strategies. Thus, it is important that the family understand that the UPSAV intervention will enable their older relative to maintain their independence. Our findings demonstrate the importance of the UPSAV intervention in older people and the population targeted to UPSAV’s intervention. Early screening of older people followed home would delay the decline of their independence.

## Data Availability

The datasets analysed during the current study are not publicly available. In this article we have dealt with personal data of natural persons. As a result, we do not have the right to share our personal data with third parties. However, they are described in the manuscript. But are available from the corresponding author on reasonable request.

## Abbreviations

ADL: Activities of Daily Living
AGGIR: Autonomie Gérontologique Groupes Iso-Ressources
BIC: Bayesian Information Criterion
BMI: Body mass index
CFAD: Constantly Functional Autonomy Decline
CI: Confidence Interval
GBTM: Group-based trajectory modeling
GDS: Geriatric Depression Scale
IADL: Instrumental activities of daily living
INSEE: Institut National de la Statistique et des Etudes Economiques
KML: K-means for Longitudinal data
MMSE: Mini-Mental State Examination
MNA: Mini Nutritional Assessment
OR: Odds Ratio
RAI: Resident Assessment Instrument
SDFA: Stable then decline functional autonomy decline
SFA: Stable Functional Autonomy
SMAF: Système de Mesure de l’Autonomie Fonctionnelle
SPPB: Short Physical Performance Battery
UPSAV: Unité de Prévention de Suivi et d’Analyse du Vieillissement
